# Design of a Kirigami Structure with a Large Uniform Deformation Region

**DOI:** 10.3390/mi12010076

**Published:** 2021-01-12

**Authors:** Hiroki Taniyama, Eiji Iwase

**Affiliations:** Department of Applied Mechanics and Aerospace Engineering, Waseda University, 3-4-1 Okubo, Shinjuku-ku, Tokyo 169-8555, Japan; taniyama@iwaselab.amech.waseda.ac.jp

**Keywords:** flexible device, stretchable electronic substrate, kirigami structure, mechanical metamaterials

## Abstract

We designed a kirigami structure with a particular shape at both ends to provide a large uniform deformation region when stretched. When a kirigami structure is stretched, non-deformation regions, where the regions’ cuts do not open, and non-uniform deformation regions, where the regions’ cuts are not uniformly deformed, are produced. The extent of the non-deformation and non-uniform deformation regions increases in proportion to the number of cut cycles in the width direction *n*_w_ this reduces the percentage of the uniform deformation region. We propose a method that increases the uniform deformation region in a kirigami structure by deforming the shape of the ends from a rectangle to a trapezoid when stretched. The proposed kirigami structure has separation lines at both ends that separate cuts in the width direction, and the position of contacts at both ends are moved to the center. The proposed kirigami structure has a large uniform deformation region, even when *n*_w_ is large, as evidenced by calculating the area of open cuts under stretching. The product of our study realizes a stretchable electro device with a large area, which maintains the position of evenly mounted functional elements when stretched.

## 1. Introduction

In recent years, new approaches have been introduced that deal with the structure of substrate materials to achieve stretchable electronic devices [1,2,3,4,5,6,7,8,9,10,11,12,13,14,15,16,17,18,19,20,21,22]. Among the innovative structures for substrates, kirigami structures with a larger apparent breaking strain than that of the material itself have attracted significant attention [6,7,8,9,10,11,12,13,14,15,16,17,18,19,20,21,22]. A kirigami structure is a structure with periodic linear cuts in a substrate. The stiffness and fracture strain of a kirigami structure as a device can be varied by changing the shape parameters. Shyu et al. elucidated the tendency of the stress–strain diagram by stretching a kirigami structure and analyzing the deformations of the structure using finite element modeling (FDM) [6]. Isobe and Okumura studied the relationship between the geometric parameters and rigidity of a kirigami-structured device using the balance of elastic strain energies [7]. Chen et al. used FDM to analyze the stress concentration at the edge of a cut with a kirigami structure [8]. We [9] modeled a kirigami structure as a series-connected spring. Based on the model, the stiffness of the kirigami structure could be analyzed, considering the non-uniform deformation regions that occurred at both ends during stretching [9]. Tang et al. controlled the inclination direction of the out-of-plane deformation in a kirigami structure by incorporating some of the structures into a kirigami structure [10,11]. Hwang and Bartlett designed a kirigami structure with a higher breaking strain than that of a conventional kirigami structure by providing small cuts at both ends [12]. Many applications have been proposed for applying a kirigami structure to substrate materials. Some examples include solar cells with solar tracking systems [13], bioprobes [14], stretchable heaters [15], soft deployable reflectors [16], strain sensors [17,18,19,20], and stretchable wirings [21,22]. These applications could be developed because of the high stretchability achieved by applying a kirigami structure to the substrate. However, kirigami structures can cause regions where cuts are connected to the roots to not open during stretching. Further, this phenomenon results in regions where cuts are hardly open and regions where the structure is not uniformly deformed compared to the center region. This is due to the inability of the roots of the kirigami structure to move in the width direction during stretching. Therefore, non-deformation regions with practically unopened cuts and non-uniform deformation regions with non-uniformly deformed cuts compared to the center region increase in proportion to the number of cut cycles in the width direction of the kirigami structure. In addition, the non-uniform deformation and non-uniform deformation regions reduce the percentage of the uniform deformation region. The amount of deformation in the length and width directions during stretching differs for the uniform deformation region, the non-uniform deformation regions, and the non-deformation regions. Therefore, functional elements cannot be evenly mounted on an electronic substrate with a kirigami structure. Conventionally, a kirigami structure is designed to reduce the number of cut cycles in the width direction and, thereby, increase the uniform deformation region. Therefore, it is necessary to make the number of cut cycles in the width direction sufficiently small compared to the number of cut cycles in the length direction, which makes it difficult to produce a large-area device. Previous studies have only taken an approach of reducing the number of cut cycles in the width direction to increase the percentage of the uniform deformation region and have not taken the approach of devising a structure at both ends of a kirigami structure. If we can realize a structure at both ends of a kirigami structure that can expand the percentage of the uniform deformation region, the number of cut cycles in the width direction can be increased and the area of a stretchable electronic device with the kirigami structure can be enlarged.

In this study, by developing a novel structure at both ends of the kirigami structure with a large number of cut cycles in the width direction, a large uniform deformation region was achieved with the stretched structure. The kirigami structure was separated into three regions: uniform deformation region, non-uniform deformation regions, and non-deformation regions. Thereafter, the ratio of each region to the entire area was calculated. Subsequently, the structure of both ends of the kirigami structure was designed based on the factors that cause non-uniform and non-deformation regions in addition to the geometrically defined deformation of the kirigami structure. The percentage of the uniform deformation region to the entire area was calculated using photographs when the kirigami structure was stretched. Finally, the percentage of the uniform deformation region for the proposed kirigami structure was compared with that of the conventional kirigami structure. In practice, we fabricated a light emitting diode (LED) array with inorganic LEDs mounted on a flexible electronic substrate using the proposed kirigami structure. To confirm that the elements were evenly distributed during stretching, we checked the shapes displayed by the LEDs when the fabricated LED array was stretched. In Section 2, a structure of the kirigami structure, its deformation, and the region during stretching are defined. In addition, a percentage of each region of the conventional kirigami structure when stretched was evaluated. In Section 3, we propose a structure at both ends of a kirigami structure to extend the uniform deformation region. We evaluated the structure at both ends of the kirigami structure by calculating the percentage of each region when stretched. Finally, an LED array with the proposed structure at both ends of the kirigami structure was fabricated, and the application of this research is presented. In Section 4, the conclusions of this study are provided.

## 2. Theory and Methods

### 2.1. Definition of Regions When a Kirigami Structure Is Stretched

**Figure 1:**
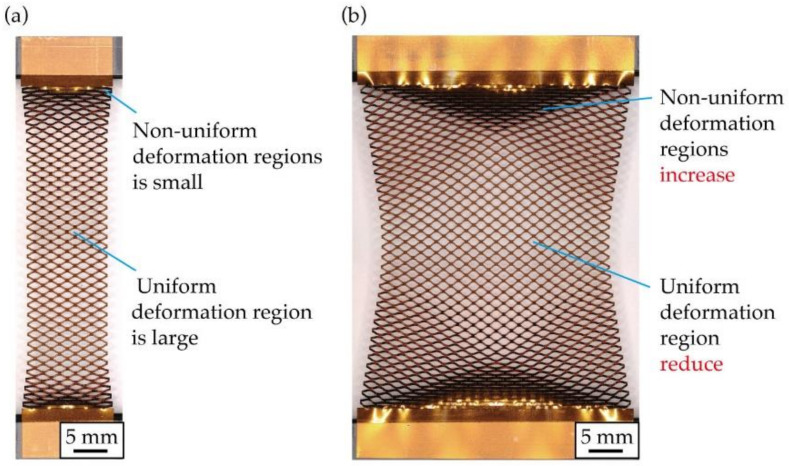
Photographs of the stretched kirigami structure: (**a**) Small number of cut cycles in the width direction, (**b**) Large number of cut cycles in the width direction.

**Figure 2:**
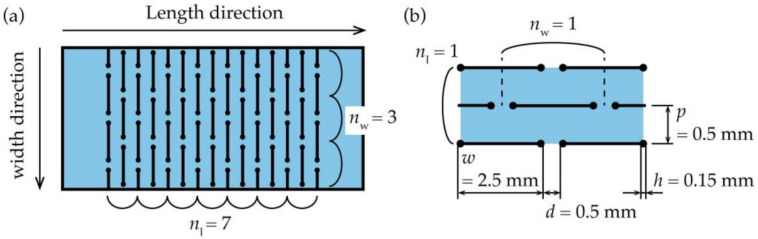
(**a**) Definition of a kirigami structure, (**b**) Definition of the dimensional parameters of a kirigami structure.

**Figure 3:**
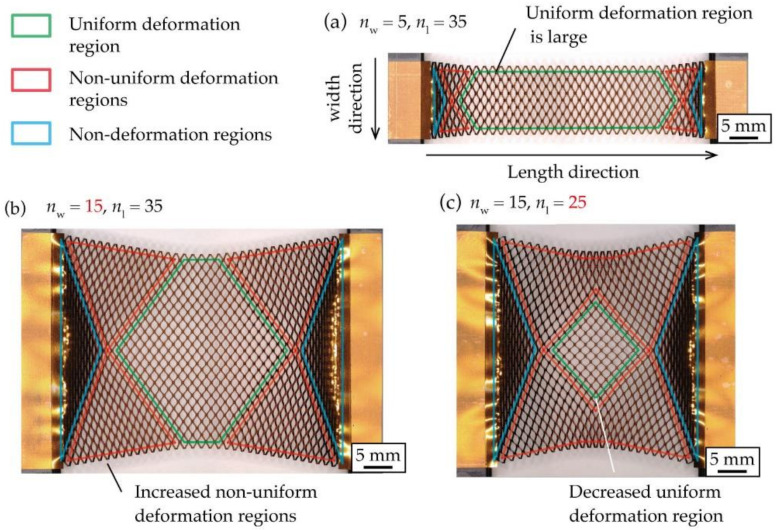
Definition of the deformed regions when stretching the kirigami structure: (**a**) *n*_w_ = 5, *n*_l_ = 35, (**b**) *n*_w_ = 15, *n*_l_ = 35, (**c**) *n*_w_ = 15, *n*_l_ = 25.

Figure 1 shows a photograph of a stretched kirigami structure. As shown in Figure 1a, when the number of cut cycles in the width direction is smaller than the number of cut cycles in the length direction, regions with non-uniform deformation and non-deformation are not noticeable. Traditionally, this has been done to increase the percentage of the uniform deformation region. Figure 1b shows a photograph of a kirigami structure when the number of cut cycles in the width direction is larger than that in Figure 1a. Figure 1b shows that as the number of cut cycles in the width direction increases, the percentage of regions that do not open cuts and have non-uniform deformation increases, and the percentage of regions that have uniform deformation decreases. This occurs because the central region contracts in the width direction during stretching, whereas contacts at the roots of the kirigami structure are fixed and cannot contract in the width direction. Increasing the number of cut cycles in the width direction of the kirigami structure increases the number of contacts in the roots. Therefore, the difference in the amount of constriction in the width direction between the center and ends is large. In addition, the percentage of the regions that do not open cuts and have non-uniform deformation increases. Figure 2 shows the definitions of a kirigami structure and shape parameters referred to in this study. The proposed kirigami structure is determined by the shape parameters shown in Figure 2. The parameters to determine the kirigami structure are the cut length, *w* [mm]; the cut distance, *d* [mm]; the cut pitch, *p* mm]; the number of cut cycles in the width direction, *n*_w_; and the number of cut cycles in the length direction, *n*_l_. A hole with a diameter *h* (mm) that is provided at the ends of a cut to inhibit crack propagation owing to stress concentration. The shape parameters of the kirigami structure used in this study were fixed at *w* = 2.5 mm, *d* = 0.5 mm, *p* = 0.5 mm, and *h* = 0.15 mm. The kirigami structure shown in Figure 2 was deformed via in-plane deformation and out-of-plane deformation. An in-plane deformation only occurs in a small strain region when the substrate to which the kirigami structure is applied is thin [7]. The kirigami structure was applied to an electronic film substrate. Therefore, we only considered the out-of-plane deformation. Figure 3 shows photographs of the kirigami structures with different *n*_w_ and *n*_l_ stretches. Three regions were defined based on the opening cuts of the incision in each photograph: the uniform deformation region, the non-uniform deformation regions, and the non-deformation regions. As shown in Figure 3a, the percentage of the uniform deformation region increases in the kirigami structure with a sufficiently small *n*_w_ compared to *n*_l_. Further, when *n*_w_ is increased to make the device larger, the percentage of the uniform deformation region is reduced, as shown in Figure 3b. Figure 3b shows that the non-deformation regions are triangular at both ends, the uniform deformation region is hexagonal in the center, and the non-uniform deformation regions constitute the rest of the regions. According to Figure 3b,c, when *n*_l_ is reduced, the percentage of the uniform deformation region is reduced. In fact, the shape of the uniform deformation region changes from hexagonal to rhombical as *n*_l_ is reduced.

### 2.2. Calculation of Regions When a Kirigami Structure Is Stretched

**Figure 4:**
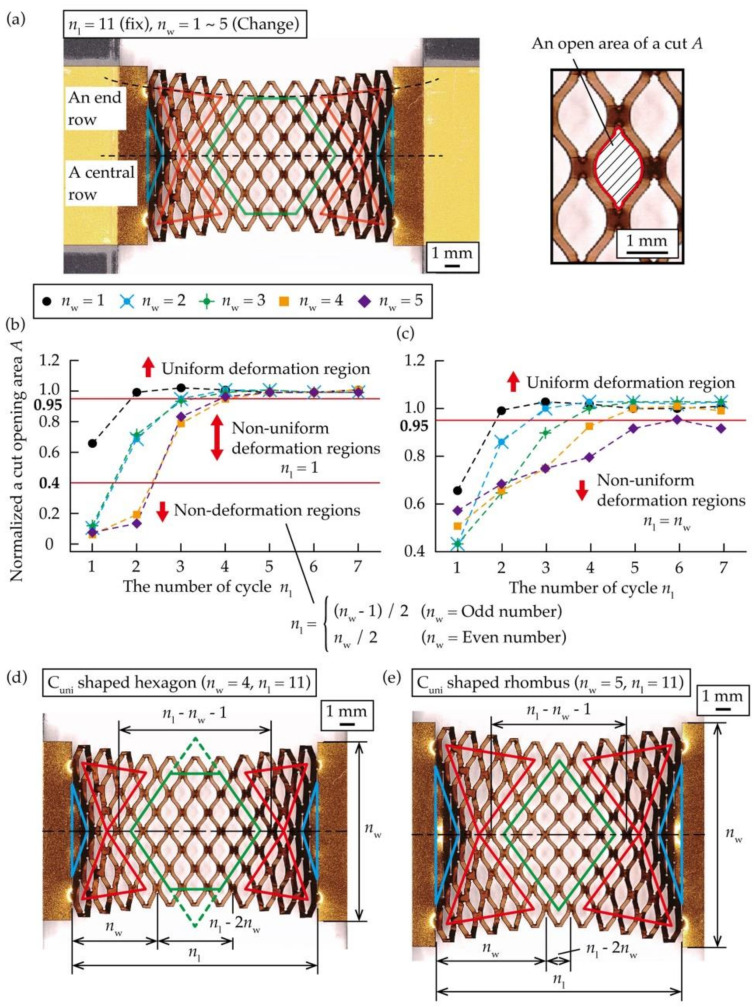
(**a**) Definition of the row measuring *A* when stretching the kirigami structure, relationship between *n*_w_ and normalized *A* when stretching the kirigami structure with a different *n*_w_: (**b**) Central row, (**c**) End row, definition of the number of cut cycles in each region when stretching the kirigami structure: (**d**) Uniform deformation region is a hexagon, (**e**) Uniform deformation region is a rhombus.

To clarify the relationship between *n*_w_ and the ratio of each region to the entire area, we calculated the position of vertices of each region when the kirigami structure with different *n*_w_ was stretched. To calculate the position of the vertices of each region shown in Figure 3, a normalized value *A* was calculated by measuring the area of open cuts of the kirigami structure under stretching deformation. The substrate material used for the test piece was a copper film with a thickness of 8 μm, deposited on a polyimide substrate with a thickness of 25 μm (Metaroiyal^®^, Toray Advanced Materials Korea, Seoul, Korea), and a film of Cu was formed via sputtering and electrolytic Cu plating. A UV laser processing machine (OLMUV-355–5A-K, Osada Photonics International, Yokote, Japan) with a wavelength of 355 nm was used to fabricate the kirigami structures on a substrate material. Five different kirigami structures with *n*_w_ = 1 to 5 were used as test objects, with a fixed *n*_l_ = 11. As shown in Figure 4a, we measured *A* in a center row and an end row of each test object when it was stretched, so that the strain *ε* was 0.5. The strain *ε* of an entire kirigami structure was calculated by dividing a given displacement by the length between the two cuts at both ends. The measured *A* is plotted on a graph where the horizontal axis is the number of cycles *n*_l_ in the length direction, and the vertical axis is *A* normalized by the central *A* in the kirigami structure. In order to normalize *A* to reduce the effect of errors during measurement and image analysis, we divided each *A* by the mean of the three central *A* in a central row when *n*_w_ was an odd number, and by the mean of the two central *A* in a central row when *n*_w_ was an even number. The created graphs are shown in Figure 4b,c. From Figure 4b,c, a range of *A* in the uniform deformation region were defined as *A* ≥ 0.95, a range of *A* in the non-uniform deformation regions were defined as 0.95 > *A* ≥ 0.40, and a range of *A* in the non-deformation regions were defined as 0.40 > *A*. From Figure 4b, the position of a vertex in the center row of the non-deformation regions was obtained by *n*_l_ = (*n*_w_ − 1)/2 when *n*_w_ was an odd number and *n*_l_ = *n*_w_/2 when *n*_w_ was an even number. The position of a vertex in the center row of the non-uniform deformation regions was found to be *n*_l_ = 1, regardless of *n*_w_. Figure 4c shows that there were no non-deformation regions in the end row, and the position of a vertex of the non-uniform deformation regions was found to be *n*_l_ = *n*_w_. Based on the above results, we calculated the percentage of the three regions to the entire region of the kirigami structure. The number of cells in an entire kirigami structure, *C*_all_, is represented by *n*_w_ and *n*_l_, as:

(1) $$C_{\mathrm{all}}=2n_{l}n_{w}-n_{l}+n_{w}-1$$

**Figure 5:**
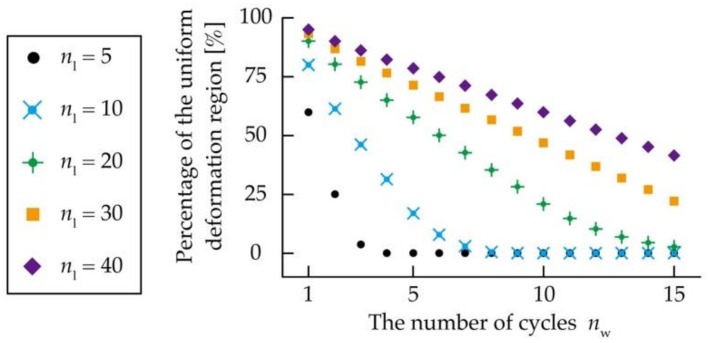
Relationship between *n*_w_, *n*_l_, and the percentage of the uniform deformation region.

The shape of the uniform deformation region varies with *n*_w_ and *n*_l_. The shape of the uniform deformation region is a hexagon for 2 *n*_w_ + 1 < *n*_l_, a rhombus for *n*_w_ + 1 < *n*_l_ ≤ 2 *n*_w_ + 1, and a non-uniform deformation region for *n*_l_ ≤ *n*_w_ + 1, as the entire center row is the non-uniform deformation and non-deformation regions. Figure 4c–e show the number of cycles when the uniform deformation region is a hexagon and a rhombus, respectively. From Figure 4d,e, the number of cells in the uniform deformation region, *C*_uni_, is described by *n*_w_ and *n*_l_, as follows:

(2) $$\begin{array}{l} C_{\mathrm{uni}}=\left\{\begin{array}{ll} {\left(n_{l}-n_{w}-1\right)}^{2}-(n_{l}-2n_{w})(n_{l}-2n_{w}-1) & \left(2n_{w}+1<n_{l}\right)\\ {\left(n_{l}-n_{w}-1\right)}^{2} & \left(n_{w}+1<n_{l}\leq 2n_{w}+1\right)\\ 0 & \left(n_{l}\leq n_{w}+1\right) \end{array}\right. \end{array}$$

where *C*_uni_ varies by *n*_w_ and *n*_l_. According to Equation (3), in the case of a hexagonal shape, the uniform deformation region, *C*_uni_, was calculated by subtracting the area of triangles of both ends from the area of the square, as shown in Figure 4d. In the case of a rhombus shape, *C*_uni_ was calculated using the number of cells in a square, as shown in Figure 4e. From Figure 4d,e, the number of cells in the non-deformation regions, *C*_non_def_, is described by *n*_w_, as

(3) $$C_{\mathrm{non}\_\mathrm{def}}=n_{w}\left(n_{w}-1\right)\;\;\;\;\;\;\;\;\;(n_{w}-1<n_{l})$$

where *C*_non_def_ varies by *n*_w_ only. According to Equation (3), for *n*_l_ ≤ *n*_w_ − 1, all cuts in the center row become non-deformation regions, and the kirigami structure can hardly be stretched. Therefore, they were not considered. From Equations (1)–(3), the number of cells in the non-uniform deformation regions, *C*_non_uni_, is described by *C*_all_, *C*_uni_, and *C*_non_def_, as

(4) $$C_{\mathrm{non}\_\mathrm{uni}}=C_{\mathrm{all}}-C_{\mathrm{uni}}-C_{\mathrm{non}\_\mathrm{def}}$$

where *C*_non_uni_ varies by *n*_w_ and *n*_l_. From Equations (1) and (2), Figure 5 shows the relationship between *n*_l_, *n*_w_, and the percentage of the uniform deformation region of the kirigami structure. In addition, as the number of cycles increases for both *n*_w_ and *n*_l_, the percentage of the uniform deformation region increases as well. Considering *n*_w_ = 10, the percentage of the uniform deformation region is as follows. When *n*_w_ to *n*_l_ is 1:1 and *n*_l_ = 10, the ratio is 0 %; when *n*_w_ to *n*_l_ is 1:2 and *n*_l_ = 20, the ratio is 20.8%; when *n*_w_ to *n*_l_ is 1:3 and *n*_l_ = 30, the ratio is 46.8%; and when *n*_w_ to *n*_l_ is 1:4 and *n*_l_ = 40, the ratio is 59.9 %. Consequently, it was established that *n*_l_ needs to be larger than *n*_w_ to increase the uniform deformation region of a conventional kirigami structure, and *n*_l_ should be approximately four times larger than *n*_w_ to increase the percentage of the uniform deformation region to more than 50%.

## 3. Results and Discussion

### 3.1. Design of the Proposed Structure at Both Ends of the Kirigami Structure to Increase the Uniform Deformation Region

**Figure 6:**
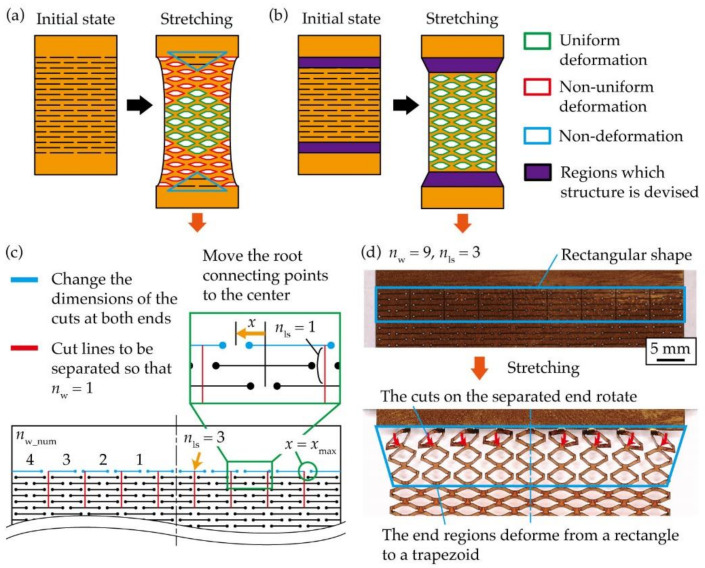
Schematic diagrams of the kirigami structure during stretching: (**a**) Conventional kirigami structure, (**b**) kirigami structure with the proposed structures at both ends, (**c**) Proposed structure at both ends of the kirigami structure to expand the uniform deformation region, (**d**) Photographs of the kirigami structure with the proposed structure at both ends during the initial state and stretching.

A structure was proposed to be applied at both ends of the kirigami structure to increase the uniform deformation region during stretching. As shown in Figure 6a, when the kirigami structure is stretched, the contact points at the roots of the kirigami structure cannot move. Therefore, the amount of constriction in the width direction differs between the center and both ends, resulting in non-uniform deformation and non-deformation regions. If the difference in width constriction between the center and ends of the kirigami structure can be reduced, the uniform deformation region can be increased. If both ends of the kirigami structure can undergo shear deformation during stretching, where they only extend in the width direction, the difference in the amount of contraction in the width direction can be reduced. However, even if in-plane shear deformation can be achieved, it is difficult to achieve shear deformation at both ends of the structure because the out-of-plane deformation causes the entire substrate to tilt. Therefore, we considered a method of transforming both ends from a rectangular to a trapezoidal shape during stretching, as shown in Figure 6b. Therefore, the difference in width constriction between the center and ends can be reduced by deforming the ends of the kirigami structure from a rectangle to a trapezoidal shape. In a case of the conventional kirigami structure shown in Figure 6a, the stress distribution gradually becomes uniform from both ends, as in the case of general materials, and cuts are uniformly deformed near the center. In a case of the kirigami structure with the newly designed structure at both ends, shown in Figure 6b, the stress distribution is non-uniform only in the designed structure regions at both ends and becomes uniform in the other regions, resulting in a uniform deformation of cuts. Figure 6c shows the structure at both ends that can be transformed from a rectangular to a trapezoidal shape when stretched. First, to open a cut of the non-deformation regions at both ends during stretching, cut lines were provided to separate the widthwise cuts periodically so that *n*_w_ = 1 at both ends. We defined the number of cycles to be separated as *n*_ls_, as shown in Figure 6c. Second, we considered moving the position of the contacts to the center to encourage the deformation of the shape at both ends from a rectangle to a trapezoid. As shown in Figure 6c, the positions of the contacts at both ends were moved to the center. Figure 6d shows photographs of the kirigami structure with the proposed structure at both ends during the initial state and stretching. The number of cut cycles in the kirigami structure is *n*_ls_ = 2, with *n*_l_ = 35 and *n*_w_ = 9. Figure 6d shows that the separation line encourages the opening of a cut of the non-deformation regions. In addition, Figure 6d shows that the position of the contacts is moved to the center, which encourages a row of ends to rotate and deform into a trapezoid. From the above results, we have proposed a structure at both ends that allows the shape of the kirigami structure to be transformed from a rectangular to a trapezoidal shape during stretching.

**Figure 7:**
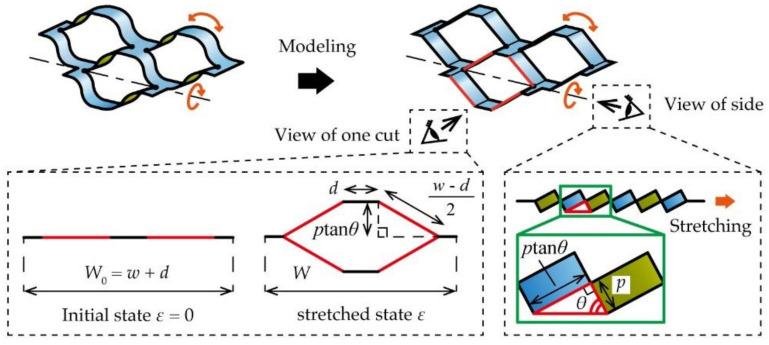
Model considering the kirigami structure as a combination of a rigid body and a pin joint.

Subsequently, we geometrically defined the deformation under elongation by modeling the kirigami structure to calculate the displacement *x* [mm] of the contact, as shown in Figure 6c. *x* is equal to the amount of movement owing to the width contraction of the kirigami structure when it was stretched such that *ε*. Figure 7 shows a model that considers the kirigami structure as a combination of a rigid body and pin joint. On that basis, a constriction in the width direction is defined geometrically. From Figure 7, it can be observed that the substrate tilted owing to out-of-plane deformation when the kirigami structure was stretched. We defined the local tilt angle in this case as *θ* [rad]. It is known that *θ* is a parameter that varies with the strain of the stretched kirigami structure only, regardless of the dimensional parameters [13]. *θ* is described by *ε* as follows:

(5) $$\theta =a\mathrm{rccos}\frac{1}{\varepsilon +1}$$

where *θ* is increased with an increase in *ε* and converges to *θ* = п. As shown in Figure 7, the width *W* [mm] of the kirigami structure is represented using *θ* and the shape parameters of the kirigami structure. *W* is described by the shape parameters of the kirigami structure as follows:

(6) $$W=2\left(d+\sqrt{{\left(\frac{w-d}{2}\right)}^{2}-{\left(p\tan\theta \right)}^{2}}\right)$$

where *W* is reduced with an increase in *θ*. As shown in Figure 6c, we defined *n*_w_num_, which is the number of contacts of the kirigami structure counting from the center. The *x* at some *n*_w_num_ is described in Figure 7 and Equation (6):

(7) $$x=n_{w\_\mathrm{num}}\left(w+d-W\right)$$

where *x* increases as *ε* increases. As shown in Figure 6c, there is a limited amount of movement *x*_max_ [mm] in a contact movement *x* because of the separate line at both ends of the kirigami structure. *x*_max_ is described by the shape parameters of the kirigami structure as follows:

(8) $$x_{\max}=\frac{w}{2}$$

where *x*_max_ is varied only with *w*. We set *x* = *x*_max_ when *x* in Equation (7) was larger than *x*_max_ in Equation (8).

### 3.2. Evaluation of the Kirigami Structure with the Proposed Structure at Both Ends

**Figure 8:**
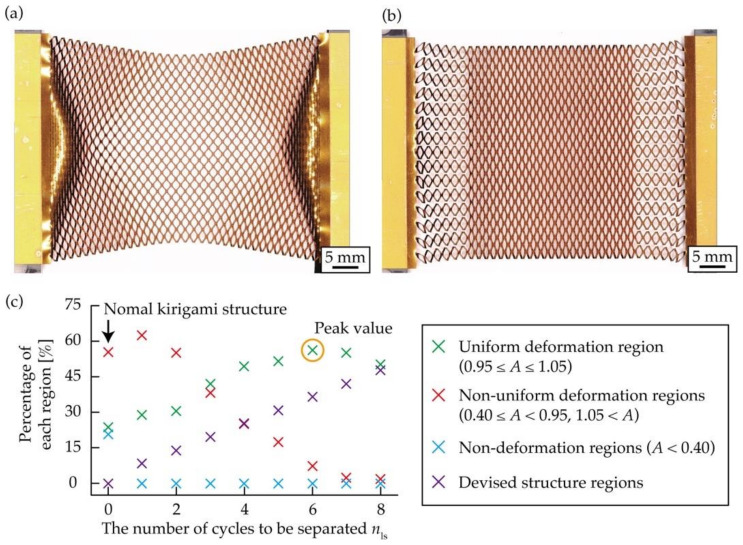
Photographs of the kirigami structures during stretching: (**a**) Conventional kirigami structure, (**b**) kirigami structure with the proposed structure at both ends, (**c**) Relationship between *n*_ls_ and the ratio of each deformation regions.

This subsection evaluates the performance of the kirigami structure with the proposed structure at both ends, as shown in Figure 6c. We measured the amount of increase in the uniform deformation region of the kirigami structure with the proposed structural change at both ends compared to the conventional kirigami structure. From Equation (7), when *n*_w_ increases, the value of *x* at the end of a row at both ends increases, and finally becomes *x*_max_. Consequently, we considered increasing *n*_ls_ when *n*_w_ was large. Therefore, we considered that the percentage of each deformed region changes with *n*_ls_. A few test objects were fabricated with different kirigami structures and *n*_ls_. Subsequently, the percentage of each deformation region was calculated by measuring *A* at *ε* = 0.5. The number of cut cycles of the kirigami structure in the test object was set to *n*_l_ = 35 and *n*_w_ = 15. The positions of the contacts at both ends were designed based on Equations (7) and (8), assuming that they were stretched so that *ε* = 0.5. Nine different test objects, numbered from 0 to 8, were prepared, where *n*_ls_ = 0 refers to the conventional kirigami structure. Figure 8a,b show photographs of the conventional and designed kirigami structures stretched to *ε* = 0.5. By comparing Figure 8a,b, it becomes clear that the proposed kirigami structure has a larger uniform deformation region. Figure 8c shows a graph where the percentage of each deformation region is plotted on the vertical axis and the *n*_ls_ on the horizontal axis. Figure 8c shows that no non-deformation regions appeared in the proposed kirigami structure. As *n*_ls_ increased, the percentage of the uniform deformation region increased, peaked at *n*_ls_ = 6, and then decreased. The reason for this pattern is the monotonic increase in the uniform deformation region in the proposed structure at both ends with increasing *n*_ls_, and the decrease in the non-uniform deformation regions, which converges to 0%. The uniform deformation region was 23.7% for the conventional kirigami structure and 56.3% for the proposed kirigami structure of *n*_ls_ = 6, which is the optimal *n*_ls_. The uniform deformation region was increased by a factor of 2.38. Therefore, it is clear that it is not enough to simply increase the size of *n*_ls_, and it is necessary to provide an optimal *n*_ls_ for a given *n*_w_.

To provide an optimal *n*_ls_ for a given *n*_w_, we calculated the percentage of the uniform deformation region for varying *n*_ls_ by fabricating the kirigami structure with multiple *n*_w_. The number of cut cycles of the kirigami structure in the test objects was fixed at *n*_l_ = 35, *n*_w_ = 15, 13, 11, 9, and 7. The positions of the contacts at both ends were designed based on Equations (7) and (8), assuming that they were stretched so that *ε* = 0.5. The percentage of the uniform deformation region was calculated for each test object by measuring *A* when the *n*_ls_ values were varied and stretched to *ε* = 0.5. The percentage of the uniform deformation region is presented in Table 1 when the kirigami structures were stretched so that *ε* = 0.5. Table 1 shows that the percentage of the uniform deformation region takes a peak value at a particular *n*_ls_ for each *n*_w_. In addition, the optimal *n*_ls_ was increased monotonically with the increase of *n*_w_.

**Figure 9:**
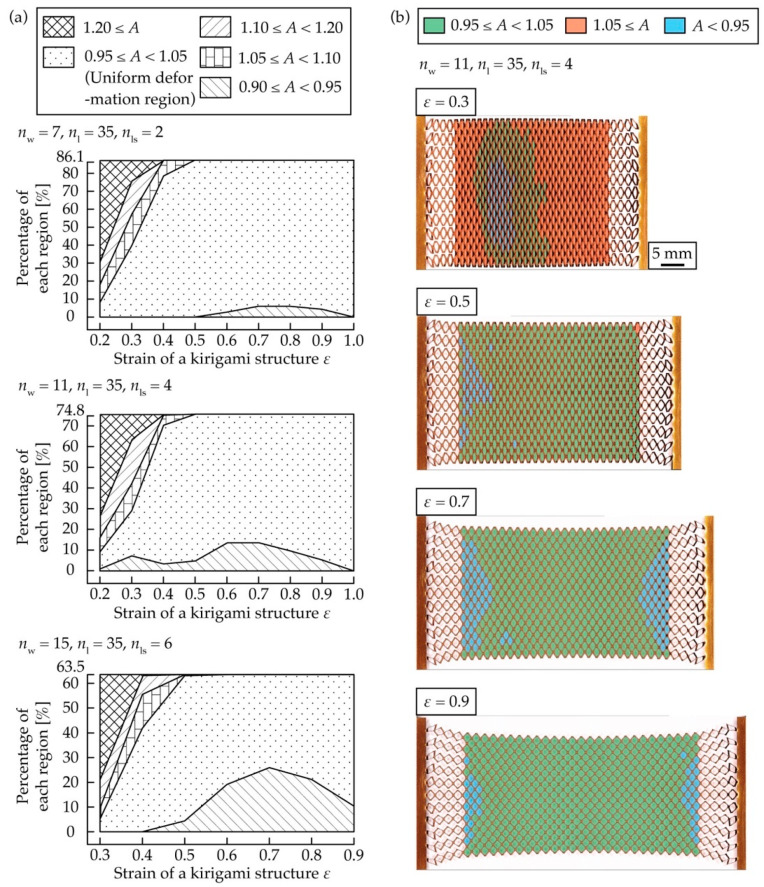
(**a**) Relationship between *ε* and the percentage of the range of each *A* relative to the entire area, (**b**) Photographs with a color histogram of the range of each *A* when stretched at *ε* = 0.3, 0.5, 0.7, and 0.9.

We checked how the uniform deformation region of the kirigami structure with an optimal *n*_ls_ for a given *n*_w_ changes with *ε* during stretching. The number of cut cycles of the kirigami structure in a test object was set to *n*_w_ = 7, *n*_ls_ = 2, *n*_w_ = 11, *n*_ls_ = 4, *n*_w_ = 15, and *n*_ls_ = 6, with the optimal *n*_ls_ according to Table 1, with *n*_l_ = 35 as the fixed number of cut cycles. The positions of the contacts at both ends were designed based on Equations (7) and (8), assuming that they were stretched so that *ε* = 0.5. The kirigami structure was stretched from *ε* = 0.2 to 1.0, and *A* was measured for every *ε* = 0.1. Figure 9a shows a graph of plotted measured values, where the horizontal axis is *ε* and the vertical axis is the percentage of the range of each *A* relative to the entire area. For the test object with *n*_w_ = 15 and *n*_ls_ = 6, measurement values were not available for *ε* = 0.2 because cuts did not open, except for the proposed structure, and for *ε* = 1.0 because the test object broke down.

From Figure 9a, for all test pieces, the percentage of the uniform deformation region has a low value up to *ε* = 0.3, which is less than half of the design value of *ε* = 0.5, and is similar to the design value of *ε* = 0.5 from *ε* = 0.4 to *ε* = 1.0. This is due to the fact that the range of 1.10 ≤ *A* is larger when *ε* = 0.2 and 0.3. Figure 9b shows photographs with a color histogram of the range of each *A* for the test pieces with *n*_w_ = 11 and *n*_ls_ = 4 at *ε* = 0.3, 0.5, 0.7, and 0.9. The decrease in the percentage of the uniform deformation region to *ε* = 0.3 was probably because the regions with the proposed structure at both ends were stretched compared to the central region, and the difference in the amount of the deformation of cuts was large, as can be seen in the photograph for *ε* = 0.3 in Figure 9b. The easy deformation of the structural devising region was due to the fact that cut lines were provided to periodically separate the widthwise cuts so that *n*_w_ = 1 at both ends. Excessive contraction in the width direction occurred in the region with the designed structure at both ends compared to the center. As a result, cuts connected to the region with the designed structure at both ends were deformed more than those in the center, and the amount of deformation gradually decreased as it approached the center, as shown in Figure 9b. As *ε* increased, the percentage of the uniform deformation region increased because the difference in the amount of deformation between the cuts in the region with the designed structure at both ends and the central region became smaller. Figure 9b shows that at *ε* = 0.7 and 0.9, the amount of deformation of the cuts at both ends connected to the region with the designed structure at both ends was smaller than that of the central cuts. However, the difference in *A* is not a big problem since it was only a few percent, as shown in Figure 9a. Figure 9a,b show that the designed kirigami structure was stretched so that *ε* = 0.5 did not expand the uniform deformation region in the small strain region up to approximately *ε* = 0.3; however, it did expand the uniform deformation region in the strain region to greater than *ε* = 0.4. The results show that the deformation should be stretched at a higher *ε* than the design value because the percentage of the uniform deformation region becomes extremely small below the design value of *ε*.

To confirm an optimal *n*_ls_ for the change in *ε*, we calculated the percentage of the uniform deformation region for varying *n*_ls_ by stretching the kirigami structure to various *ε*. The number of cut cycles of the kirigami structure in the test objects was fixed at *n*_l_ = 35 and *n*_w_ = 7. The positions of the contacts at both ends were designed based on Equations (7) and (8), assuming that they were stretched so that *ε* = 0.3, 0.5, 0.7, and 0.9. The percentage of the uniform deformation region was calculated for each test object by measuring *A* when the *n*_ls_ values were varied and stretched to *ε* = 0.3, 0.5, 0.7, and 0.9. The corresponding percentage of the uniform deformation regions is presented in Table 2. The measurement error of *A* at *ε* = 0.3 was large, so that three measurements were made on different test pieces at *ε* = 0.3, and the average values are shown in Table 2; the optimal *n*_ls_ does not change when *ε* is changed and *n*_w_ = 7.

**Figure 10:**
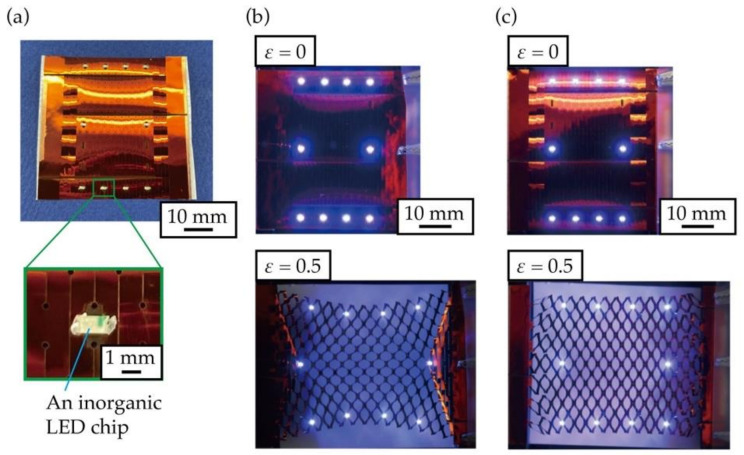
Photographs of the fabricated light emitting diode (LED) array: (**a**) Fabricated LED array when stretched, (**b**) LED array with the conventional kirigami structure, (**c**) LED array with the proposed kirigami structure.

Finally, to demonstrate the usefulness of the designed kirigami structure at both ends, we fabricated an LED array with inorganic LEDs mounted on a substrate with the designed kirigami structure applied to a film-like electronic substrate. Polyimide copper substrates were used as the substrate material. The kirigami structure and copper wiring patterning were performed using a UV laser processing machine. The dimensions of the kirigami structure were *w* = 5.5 mm, *d* = 1.6 mm, *p* = 1.2 mm, and *h* = 0.15 mm. The number of cut cycles in the kirigami structure was set to *n*_l_ = 15, *n*_w_ = 7, and the optimal *n*_ls_ from Table 1 was *n*_ls_ = 2. The positions of the contacts at both ends were designed based on Equations (7) and (8), assuming that they were stretched so that *ε* = 0.5. We fabricated an LED array using inorganic LEDs on an electronic substrate with a conventional kirigami structure and the proposed kirigami structure. Further, the array was in the form of a rectangle shape. Photographs of the fabricated LED array are shown in Figure 10a. Photographs of the fabricated LED arrays during stretching are shown in Figure 10b,c. Figure 10b shows that the rectangular shape made by the LEDs was distorted when the LED array was stretched in the conventional kirigami structure. In contrast, in the designed kirigami structure, shown in Figure 10c, the rectangular shape represented by the LEDs was not distorted during stretching. Therefore, the designed kirigami structure placed the mounted functional elements evenly and uniformly, even when stretched. In summary, it was shown that the designed kirigami structure was useful for stretchable displays and sensor arrays where sensors need to be spaced evenly during stretching.

## 4. Conclusions

We designed a kirigami structure with a novel structure at both ends that had a large uniform deformation region when stretched. Based on photographs of the stretched kirigami structure, we defined three deformation regions: uniform deformation regions, non-uniform deformation regions, and non-deformation regions. To clarify the relationship between the percentage of the three defined regions and the *n*_w_ of the kirigami structure, we measured *A* of stretched kirigami structures with different values for *n*_w_. As a result, we established that the percentage of uniform and non-uniform deformation regions varied with *n*_w_ and *n*_l_, and the percentage of non-deformation regions varied with *n*_w_. In addition, we discovered that in the conventional kirigami structure, the number of *n*_l_ to *n*_w_ was approximately four times larger than that of *n*_w_; hence, the percentage of the uniform deformation region was more than 50%. Subsequently, to achieve a stretched kirigami structure with a large uniform deformation region, we proposed a particular design for the structure at both ends of the kirigami structure. The proposed kirigami structure had separation lines at both ends such that *n*_w_ = 1, and the position of contacts at both ends were moved to the center. To check whether the percentage of the uniform deformation region was increased owing to the proposed structure at both ends, the percentage of the uniform deformation region was calculated and compared with that of the conventional kirigami structure. It was confirmed that the uniform deformation region of the proposed kirigami structure was larger than that of the conventional kirigami structure during stretching by approximately 2.38 times. We checked how the uniform deformation region of the kirigami structure with an optimal *n*_ls_ for a given *n*_w_ changed with ε during stretching. The results show that the proposed kirigami structure should be stretched at a higher *ε* than the design value because the percentage of the uniform deformation region becomes extremely small below this value. Finally, we fabricated an LED array with inorganic LEDs mounted on an electronic substrate with the designed kirigami structure. The shapes of the LEDs were not distorted when the fabricated LED arrays were stretched, indicating that the designed kirigami structure could evenly and uniformly distribute the mounted functional elements, even when stretched.

By applying the proposed structure at both ends of a kirigami structure, it was possible to realize a kirigami structure with a large uniform deformation region when *n*_w_ was large, which could not be realized in previous studies. The product of our study further enables to evenly mount functional elements on an electronic substrate with a large device area, which allows one to create stretchable displays and sensor arrays where functional elements need to maintain their position when stretched.

## Figures and Tables

**Table 1:** Percentage of the uniform deformation region in the proposed kirigami structures with various *n*_w_ and *n*_ls._

*n*_ls_	*n*_w_ = 15	*n*_w_ = 13	*n*_w_ = 11	*n*_w_ = 9	*n*_w_ = 7
1	28.9%	38.6%	50.7%	64.2%	77.9%
2	30.7%	50.3%	55.6%	81.3%	80.3%
3	42.1%	56.9%	66.8%	80.3%	79.8%
4	49.7%	58.1%	74.1%	73.8%	74.4%
5	51.7%	66.7%	68.3%	68.3%	\
6	56.3%	62.6%	62.1%	\	\
7	55.4%	56.8%	\	\	\
8	50.3%	\	\	\	\

**Table 2:** Percentage of the uniform deformation region in the proposed kirigami structures with the designed *x* at both ends for various *ε* and *n*_ls_.

*n*_ls_	*ε* = 0.3	*ε* = 0.5	*ε* = 0.7	*ε* = 0.9
1	59.8%	77.9%	77.9%	breaking
2	65.8%	80.3%	83.9%	85.0%
3	51.4%	79.8%	80.3%	80.5%
4	31.0%	74.4%	74.8%	74.8%
